# In Silico Studies of C_**3**_ Metabolic Pathway Proteins of Wheat (*Triticum aestivum*)

**DOI:** 10.1155/2013/294759

**Published:** 2012-12-26

**Authors:** Muhammad Kashif Naeem, Sobiah Rauf, Hina Iqbal, Muhammad Kausar Nawaz Shah, Asif Mir

**Affiliations:** ^1^Department of Plant Breeding and Genetics, PMAS-Arid Agriculture University, Rawalpindi, Pakistan; ^2^Department of Bioinformatics and Biotechnology, FBAS, International Islamic University, Islamabad, P.O. Box 44000, Pakistan

## Abstract

Photosynthesis is essential for plant productivity and critical for plant growth. More than 90% of plants have a C_3_ metabolic pathway primarily for carbon assimilation. Improving crop yields for food and fuel is a major challenge for plant biology. To enhance the production of wheat there is need to adopt the strategies that can create the change in plants at the molecular level. During the study we have employed computational bioinformatics and interactomics analysis of C_3_ metabolic pathway proteins in wheat. The three-dimensional protein modeling provided insight into molecular mechanism and enhanced understanding of physiological processes and biological systems. Therefore in our study, initially we constructed models for nine proteins involving C_3_ metabolic pathway, as these are not determined through wet lab experiment (NMR, X-ray Crystallography) and not available in RCSB Protein Data Bank and UniProt KB. On the basis of docking interaction analysis, we proposed the schematic diagram of C_3_ metabolic pathway. Accordingly, there also exist vice versa interactions between 3PGK and Rbcl. Future site and directed mutagenesis experiments in C_3_ plants could be designed on the basis of our findings to confirm the predicted protein interactions.

## 1. Introduction 

Photosynthesis is arguably the most important energy conversion process on earth because the chemical energy it yields is the base of food chains that sustain the overwhelming majority of other life forms. Plants utilize atmospheric CO_2_ to liberate oxygen and synthesize carbohydrates during photosynthesis. It is an event where radiant energy of sunlight is utilized to convert carbon dioxide into photosynthetic by-products. On the basis of 1st product of photosynthesis or modifications in Calvin-Benson Cycle plants are grouped into three categories: C_3_ plants, C_4_ plants, and CAM plants. More than 90% of plants have C_3_ metabolic pathway primarily for the carbon assimilation, whereas only 3% plants utilized C_4_ metabolic pathway [1], and crassulacean acid metabolism (CAM) is found in sixteen thousand species of plants [2]. 

C_3_ plants are less photosynthetically efficient and cannot grow in hot areas because of the activity of *RuBisCO* enzyme. RuBisCo has the ability to fix both carbon dioxide and oxygen that result in the loss of carbon, nitrogen, and energy from plants [3]. *Bread wheat *(*Triticum aestivum* L.), durum wheat (*Triticum turgidum* L.), and barley (*Hordeum vulgare* L.) are the most outstanding C_3_ crops in terms of cultivated area and food source, from the Holocene time up to present [4]. Improving crop yields for food and fuel is a major challenge for plant biology which is necessary to meet the requirements of a rapidly increasing world population [5]. Wheat is a common staple cereal food crop in all parts of the world and contributes to 28% of the world's edible dry matter (DM) and up to 60% of the daily calorie intake in several developing countries [6].

To enhance the production of wheat necessitates adopting strategies that can create the change in plants at the molecular level through one of two approaches: wet lab or computational. The study utilized computational bioinformatics and interactomics analysis of C_3_ metabolic pathway proteins in wheat. Structural bioinformatics is concerned with the prediction, analysis, and visualization of the 3D structure of proteins. Besides NMR and X-ray Crystallography that entail enormous experimental costs, time, and laborious procedure, another recent and attractive approach built for the analysis of protein structure has recently emerged in structural bioinformatics. There are three approaches primarily utilized to predict 3D structures of studied proteins: (1) homology modeling or comparative modeling [7, 8], (2) threading or fold recognition [9–11], and (3) ab-initio prediction [12–15]. Proteins interact with each other as well as with other macromolecules to accomplish their functions within cell; therefore protein-protein interactions are complex and play a crucial role in most biological processes to determine the actual functioning of protein. Wet lab approaches such as tandem affinity purification mass spectrometry [16], yeast two hybrid [17, 18], and some others are found to enable the mapping of complex protein interactions. It is difficult to take advantage of these experimental techniques due to the complexity of biological molecules compared to computational protein-protein docking that offer more benefits [19]. More recently, a high-throughput docking approach for protein-protein interaction has been reported [20]. The aim of this study is to improve the understanding of C_3_ metabolic pathway proteins through in silico analysis and to study protein interaction in wheat. Each protein have has an important role, either in CO_2_ fixation to regenerate ribulose 1, 5-bisphosphate or to synthesize starch and sucrose. This study selected only nine of proteins involved in the C_3_ Pathway. The nine selected protein structures have not yet been determined through wet lab experiment (NMR, X-ray Crystallography) nor available in RCSB Protein Data Bank and UniProt KB.

## 2. Materials and Methods 

**Figure 1:**
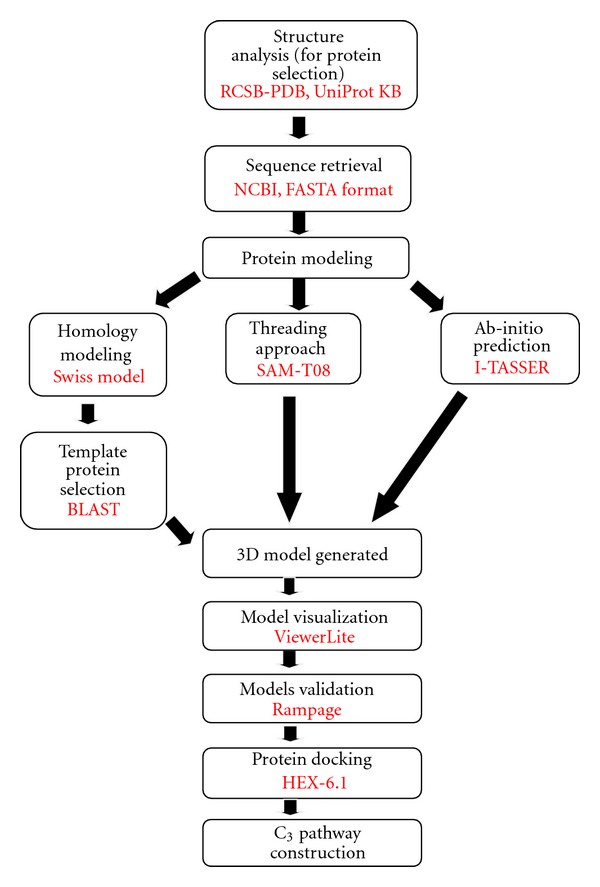
A flowchart representing the use of tools in a sequential manner for current study model.

A flowchart representing the use of tools in a sequential manner for study of nine important proteins, involved in C_3_ metabolic pathway of wheat, is given in Figure 1. Sequences of these proteins were retrieved from *NCBI *(*National Center for Biotechnology Information*) in *FASTA* format, and it was used as a query for further analysis [21].

In order to generate three-dimensional models homology, threading and ab-intio approach was applied.

*Homology Modeling.* The modeling of the 3D structure of the proteins was executed by *Swiss-Modeler *(http://swissmodel.expasy.org/) program [22, 23]. 

*Threading Approach. SAM-T08* (http://compbio.soe.ucsc.edu/SAM_T08/T08-query.html) [24]. SAM-T08 web-based program for the modeling of three-dimensional structures of all selected protein sequences was used. 

*Ab-Initio Prediction. Iterative Threading Assembly Refinement *(*I-TASSER)* [25, 26] online web server for predicting 3D protein structure was used.

*Model Visualization and Evaluation.* For model visualization the *ViewerLite software was used*. Rampage server (http://mordred.bioc.cam.ac.uk/~rapper/rampage.php) was used for evaluating and assessing the accuracy of the model [27, 28].

*Protein-Protein Interaction.* Protein-protein interaction is generated by the help of *HEX 6.1*. HEX 6.1 program is used to speed up docking estimations; the method provides a feasible orientation rapidly and precisely [29]. 

## 3. Results

### 3.1. Retrieval of Target and Recognition of Template Protein

**Figure 2:**
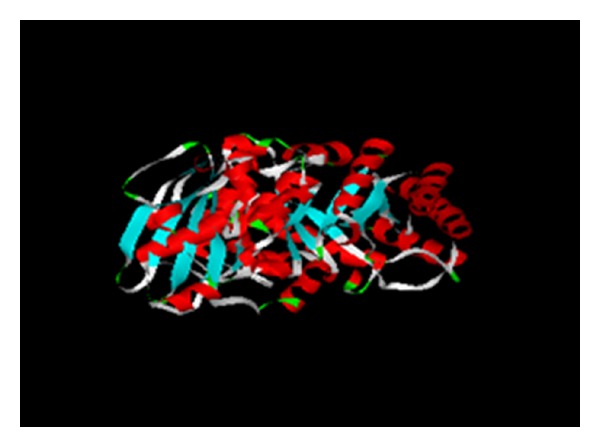
SAM-T08 model of Rbcl and visualized by ViewerLite. Red representing alpha helix, sky blue representing beta sheet, and green representing loop in model.

**Figure 3:**
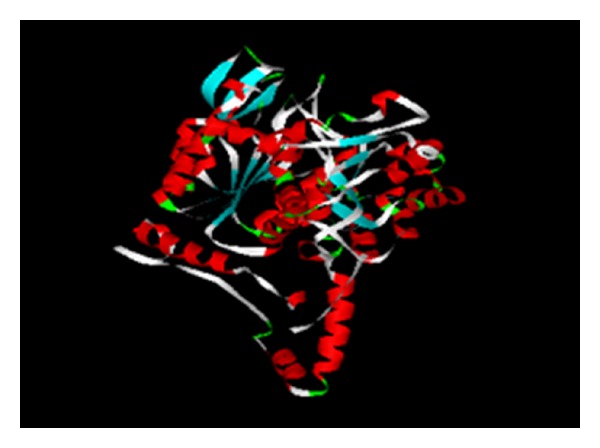
SAM-T08 model of 3PGK and visualized by ViewerLite. Red representing alpha helix, sky blue representing beta sheet, and green representing loop in model.

**Figure 4:**
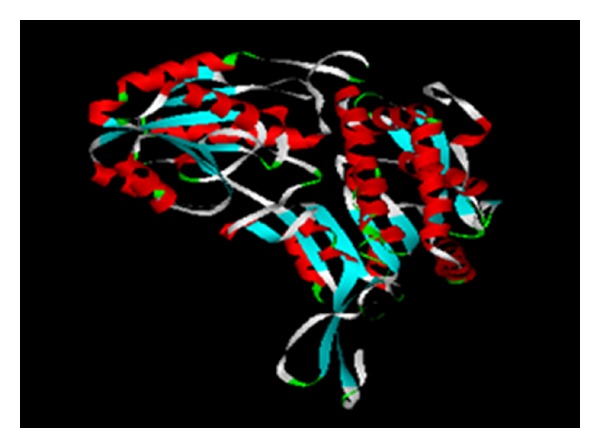
SAM-T08 model of GAPN and visualized by ViewerLite. Red representing alpha helix, sky blue representing beta sheet, and green representing loop in model.

**Figure 5:**
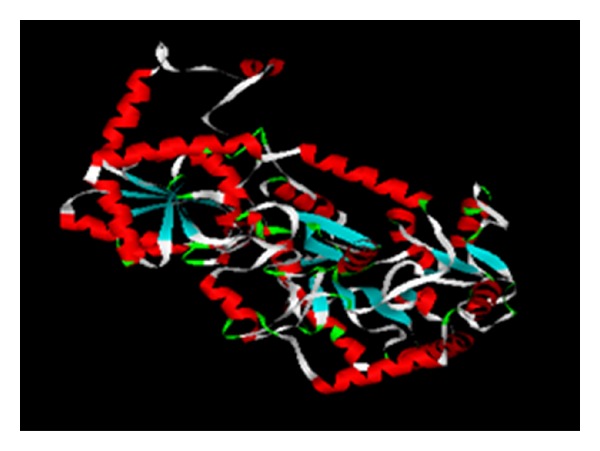
SAM-T08 model of SPS II and visualized by ViewerLite. Red representing alpha helix, sky blue representing beta sheet, and green representing loop in model.

**Figure 6:**
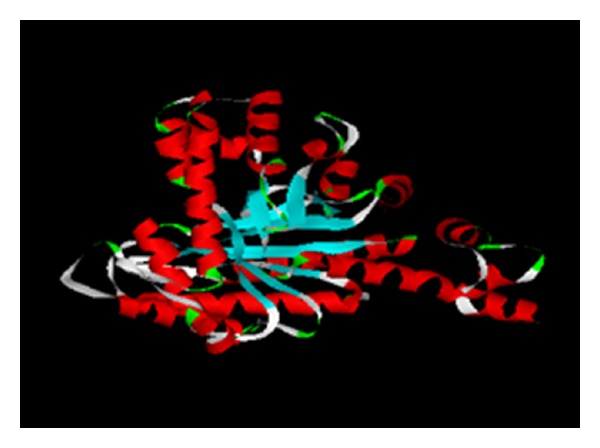
SAM-T08 model of Fba and visualized by ViewerLite. Red representing alpha helix, sky blue representing beta sheet, and green representing loop in model.

**Figure 7:**
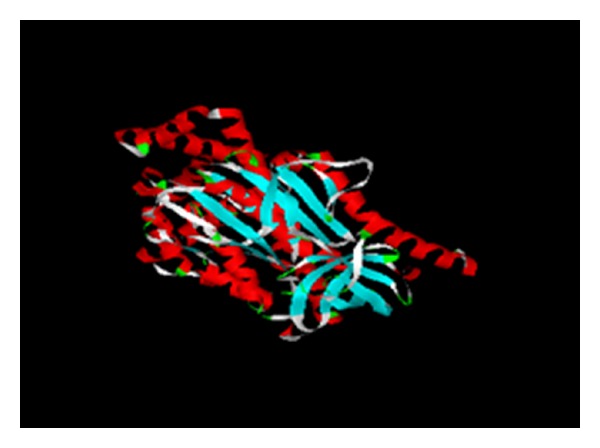
SAM-T08 model of atp A and visualized by ViewerLite. Red representing alpha helix, sky blue representing beta sheet, and green representing loop in model.

**Figure 8:**
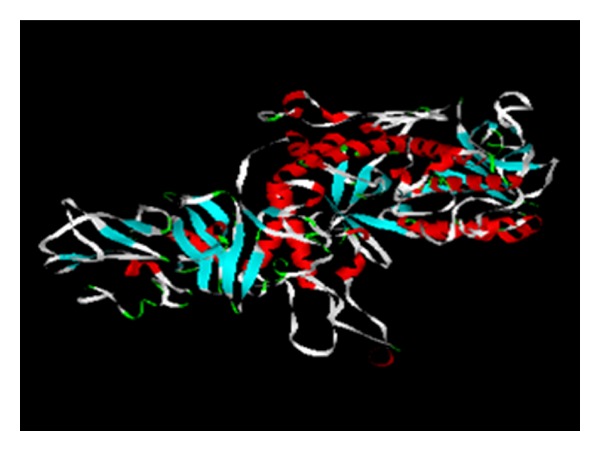
I-TASSER model of Sbe I and visualized by ViewerLite. Red representing alpha helix, sky blue representing beta sheet, and green representing loop in model.

**Figure 9:**
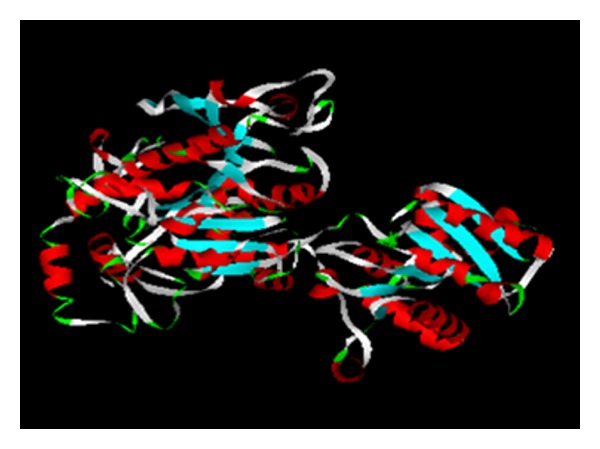
SAM-T08 model of WAXY and visualized by ViewerLite. Red representing Alpha Helix, sky blue representing Beta sheat, and green representing Loop in model.

**Figure 10:**
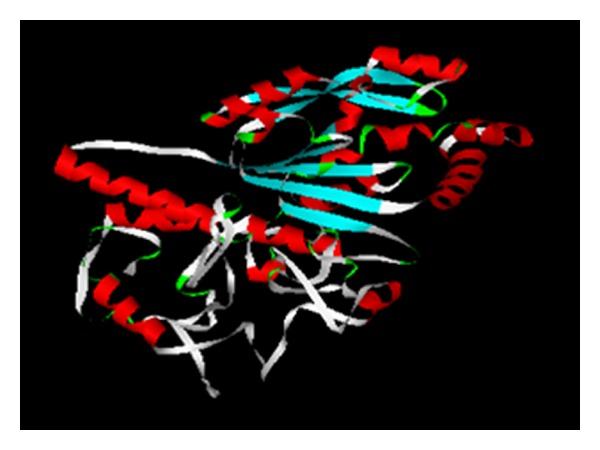
SAM-T08 model of PRK and visualized by ViewerLite. Red representing alpha helix, sky blue representing beta sheet, and green representing loop in model.

For this study, selected proteins involved in C_3_ metabolic pathway were retrieved from NCBI (having maximum residual length), and their protein sequences in FASTA were used for a query sequence similarity search. PSI-BLAST identified the potential template proteins with their respective PDB-ID with maximum similarity and *E* value. The BLAST results of nine target proteins are summarized in Table 1. As per our findings Rbcl and ATP synthase template showed 100% similarity with target sequence. GAPN and F-base demonstrated similarity more than 90%. 3PGK and WAXY showed percent similarity more than 80%, and remaining 3 proteins demonstrated the sequence similarity from 70 to 48%. After the selection of potential templates 3D model is generated by the use of Swiss-modeler program. We determined the length and alignment accuracy of generated model by Viewerlite. Five proteins (Rbcl, 3PGK, GAPN, Fba, and ATP A) showed the model length percentage more than 80%. While remaining four proteins (SPS II, Sbe I, WAXY, and PRK) have less than 80% (Table 2). All 3D full length models were generated by SAM-T08 (based on HMM) except Sbe I. Modeling of Sbe I protein is done by I-TASSER (using ab-intio approach) as shown in given figures (Figures 2, 3, 4, 5, 6, 7, 8, 9, and 10). 

### 3.2. Model Validation

Three-dimensional protein models validation is last step of protein modeling. It evaluates the significance and accuracy by using Ramachandran plot in Procheck web-based server. Results of Ramachandran plot demonstrate that eight proteins have more than 90% number of residues in favored region which shows the accuracy of these models. One protein (Sbe1) has residues in favored region <90% as 83.6% and the lower level significance of Sbe I model. Model evaluation is done by Rampage program, and results are described in Ramachandran plot (Table 2). The summary of Ramachandran plot results is presented in Table 3. ATP A protein showed maximum residues 97.8% in favored region among all protein. 3PGK protein depicted the least residues 0.3% in outlier region, and PRK protein demonstrated the maximum residues 1.8% in outlier region. 

### 3.3. Protein-Protein Interaction

Protein-protein interaction describes the actual functioning of protein and their role in metabolic pathway. We used HEX-6.1 program for all our selected proteins. Docking results for each protein are summarized in Table 4. Docking results predicted that Rbcl has strong interaction with 3PGK indicated by minimum *E* value −709.8. 3PGK showed strong linkage with two proteins that is Fba (−664.83) and GAPN (−653.19). GAPN showed minimum *E* value −621.78 and depicted strong interaction with SPS II. Our results showed that GAPN has no interaction with Rbcl, Fba, ATP A, WAXY, and PRK. Fba has strong linkage with Sbe I as shown by minimum docking value −654.16.WAXY also showed strong interaction with Sbe I (Table 4). Sbe I demonstrated the minimum value with the PRK (−600.58) depicting the strong interaction. 

## 4. Discussion 

Over 90% of plants have C_3_ metabolic pathway. C_3_ photosynthetic pathway present in major crops such as wheat, barley, and rice. Wheat is major staple food crop all over the world. Decrease in productivity of wheat due to C_3_ metabolic pathway involved oxygenation reactions and results in loss of energy 25–30%. Improve the efficiency of C_3_ metabolic pathway needs to know the functions of proteins involved in C_3_ pathway. Consequently, it is very crucial to be familiar with the 3D structure of proteins which gave insight into molecular functioning, which enhance the better understanding of physiological processes and biological systems. Therefore in our study, eight protein sequences were used and modeled via threading approach, as these proteins have >700 amino acids, and models are constructed by SAM-T08. For one protein, that is, Sbe I (830 aa in length), I-TASSER program generated full length model (Figure 8). Full length generated models are significant to use for future studies. Significance of our modeling studies for nine proteins was depicted by confirming that there is no crystalline structure present in protein databank till date.

**Figure 11:**
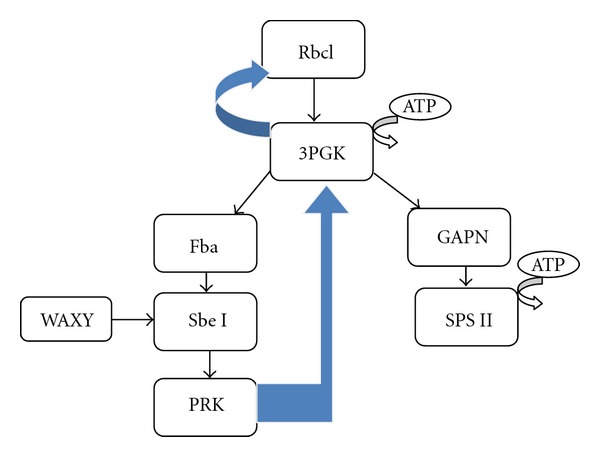
Schematic representation of C_3_ metabolic pathway proposed according to our protein-protein interaction results. The blue color arrow represents the predicted unique interactions in our model study other than existing results known in C_3_ pathway interactions.

On the basis of docking result we proposed the schematic diagram of C_3_ metabolic pathway (Figure 11). As per our analysis, Rbcl showed strong interaction with 3PGK in C_3_ metabolic pathway, as we obtained minimum docking value (Table 4), while Rbcl as receptor indicated that it has no interaction with GAPN, Fba, WAXY, and PRK. 

Our docking results also described that 3PGK as a receptor demonstrated the maximum interaction value with Rbcl. It is predicted that 3PGK regenerate the Rubisco enzyme. 3PGK showed strong linkage with two proteins, that is, Fba and GAPN. 3PGK interaction with Fba leads the process to starch biosynthesis, while its interaction with GAPN produces sucrose, as final products. Previous in vitro study depicted 3PGK (EC 2.7.2.3) and GAPN (EC 1.2.1.13) association [30].

Glyceraldehyde-3-P dehydrogenase (GAPN) is essential for plant metabolism and development. GAPN depicted strong interaction with SPS II in our analysis. SPS II are carbon compound and are indispensable for growth and development of the plant [31, 32]. In vivo it is shown that phosphorylation of SPS II changes the activity of SPS II and carbon partitioning [33]. In this regard SPS II enzyme is very essential for carbon partitioning, and also SPS II synthesize sucrose essential for plant development and growth. Sucrose formation is positively correlated with the rate of photosynthesis. We found no interactions of GAPN with Rbcl, Fba, ATP A, WAXY, and PRK. 

Our results in Table 4 demonstrated that Fba has strong linkage with Sbe I. Fba is involve in the synthesis of starch, therefore they interact with the Sbe I help in starch formation. WAXY also showed strong interaction with Sbe I. Previous studies described that in rice developing endosperm C53 fragment (WAXY gene) interacts with two ACGT elements G-box and Hex (BZIP protein or SbeI), and both genes coordinately are regulated [34]. 

Sbe I demonstrated in (Table 4) the minimum value with the PRK revealing the strong interaction comparison to other proteins. PRK docking results demonstrated that it has strong interaction with 3PGK. It depicted that PRK regenerates the phosphoglycerate kinase which is also reported in a previous study by Raines in 2011 [35]. It demonstrated that reduced activity of PRK protein was enough to slower the photosynthesis process and decrease the plant productivity [36].

Our results predict that Fba, GAPN, and PRK have least interaction with other proteins in C_3_ metabolic pathway. Similar to our results previous studies also demonstrated that GAPN, Fba, and PRK had no impact on carbon assimilation [37, 38].

## 5. Conclusion

- During the model study we have predicted the selected nine proteins structures of C_3_ pathway using bioinformatics methods as these are not determined through wet lab experiment (NMR, X-ray Crystallography) and were not available in RCSB Protein Data Bank and UniProt KB.
- According to generalized known metabolic pathway Rbcl interacts with 3PGK only. But our study showed that 3PGK also interact with Rbcl to complete the CO_2_ fixation pathway. So there also exist vice versa interactions of 3PGK with Rbcl.
- In biochemical pathways PRK interacts with Rbcl to complete the cycle, but according to our predicted pathway interaction between PRK with Rbcl is mediated through 3PGK.
- Future site directed mutagenesis experiments in C_3_ plants could be designed on the basis of our findings to confirm the predicted protein interactions.

## Figures and Tables

**Table 1:** Summary of selected nine proteins involved in C_3 _metabolic pathway of wheat and their BLAST results.

Number	Target proteins	Gene name	Gene ID number	Residues	PDB ID	% MI*	*E* value*
1	RuBisCO large subunit	RbcL	BAB 47042.1	477	4RUB_A	100%	0.0
2	Phosphoglycerate kinase	3PGK	CAA 51931.1	480	1VPE_A	80%	2*e* − 128
3	NADP-dependent glyceraldehyde-3-phosphate dehydrogenase	GAPN	Swiss Prot Q8LK61.2	496	1EUH_A	95%	1*e* − 137
4	Sucrose phosphate synthase II	SPS II	ADK 11932.1	626	2R60_A	50%	7*e* − 50
5	Fructose-bisphosphate aldolase	Fba	CBH 32613.1	385	3KX6_A	90%	1*e* − 103
6	ATP synthase alpha subunit	ATP A	AAA 84725.1	504	1FX0_A	100%	0.0
7	Starch branching enzyme I	Sbe1	CAA 72987.1	830	3O7Y_A	70%	4*e* − 40
8	Granule-bound starch synthase 1	WAXY	SwissProt P27736.1	615	3D1J_A	83%	1*e* − 59
9	Phosphoribulokinase	PRK	Swiss Prot P26302.1	404	2JEO_A	48%	4*e* − 16

% MI: percent maximum similarity; *E* value: energy value.

**Table 2:** Summary and comparison of ViewerLite results of selected proteins with various web servers.

Proteins	Number of residues (amino acids)	Web servers			
		Swiss-model	% model length	SAM-T08	I-TASSER
Rbcl	477	1-464	97.2%	1-477	—
3PGK	480	1-398	82.9%	1-480	—
GAPN	496	1-474	95.5%	1-496	—
SPS II	626	1-456	72.89%	1-626	—
Fba	385	1-358	92.87%	1-385	—
ATP A	504	1-477	94.6%	1-504	—
Sbe I	830	1-612	73.73%	—	1-830
WAXY	615	1-405	65.85%	1-615	—
PRK	404	1-215	53.2%	1-404	—

**Table 3:** Rampage evaluation results of studied proteins showing the accuracy of our models.

Proteins	Favored region	Allowed region	Outlier region
Rbcl	96.8%	2.7%	0.4%
3PGK	95.8%	3.9%	0.3%
GAPN	95.7%	3.3%	1.0%
SPS II	96.0%	3.0%	1.0%
Fba	96.1%	3.1%	0.8%
ATP A	97.8%	1.8%	0.4%
Sbe I	83.6%	10.4%	6.0%
WAXY	94.6%	4.6%	0.8%
PRK	96.6%	1.6%	1.8%

**Table 4:** Summary of protein docking results.

		Ligands								
		RbcL	3PGK	GAPN	SPS II	Fba	Sbe I	WAXY	PRK	ATP A
Receptor	RbcL	—	**−709.8**	0.00	−661.01	0.00	−554.51	0.00	0.00	−647.4
	3PGK	**−731.7**	—	**−653.19**	−560.03	**−664.83**	−546.61	−629.23	−601.07	−638.00
	GAPN	0.00	−697.78	—	**−621.78**	0.00	−442.61	0.00	0.00	0.00
	SPS II	−668.63	−563.23	−564.06	—	−549.66	−389.00	−497.30	−604.34	−530.92
	Fba	0.00	−646.49	0.00	−564.97	—	**−654.16**	0.00	0.00	0.00
	Sbe1	−540.88	445.36	−460.27	−387.54	−670.23	—	−489.67	**−600.58**	−412.56
	WAXY	0.00	−684.00	0.00	−509.77	0.00	**−470.18**	—	0.00	−422.82
	PRK	0.00	**−614.7**	0.00	−537.44	0.00	−528.05	0.00	—	0.00
	ATP A	−550.1	**−621.99**	0.00	**−675.29**	0.00	−483.83	−308.53	0.00	—

## References

[1] Sage RF, Monson RK (1999). *C4 Plant Biology*.

[2] Dodd AN, Borland AM, Haslam RP, Griffiths H, Maxwell K (2002). Crassulacean acid metabolism: plastic, fantastic. *Journal of Experimental Botony*.

[3] Portis AR, Parry MAJ (2007). Discoveries in Rubisco (ribulose 1,5-bisphosphate carboxylase/oxygenase): a historical perspective. *Photosynthesis Research*.

[4] Evans LT (1998). *Feeding the Ten Billion: Plants and Population Growth*.

[5] Edgerton MD (2009). Increasing crop productivity to meet global needs for feed, food, and fuel. *Plant Physiology*.

[6] Gill BS, Appels R, Botha-Oberholster AM (2004). A workshop report on wheat genome sequencing: international genome research on wheat consortium. *Genetics*.

[7] Fiser A, Do RK, Sali A (2000). Modeling of loops in protein structures. *Protein Science*.

[8] Martí-Renom MA, Stuart AC, Fiser A, Sánchez R, Melo F, Šali A (2000). Comparative protein structure modeling of genes and genomes. *Annual Review of Biophysics and Biomolecular Structure*.

[9] Xu Y, Xu D (2000). Protein threading using PROSPECT: design and evaluation. *Proteins*.

[10] David R, Korenberg MJ, Hunter IW (2000). 3D-1D threading methods for protein fold recognition. *Pharmacogenomics*.

[11] Skolnick J, Kihara D, Zhang Y (2004). Development and large scale benchmark testing of the PROSPECTOR 3.0 threading algorithm. *Proteins*.

[12] Bradley P, Misura K, Baker D (2005). Biochemistry: toward high-resolution de novo structure prediction for small proteins. *Science*.

[13] Klepeis JL, Wei Y, Hecht MH, Floudas CA (2005). Ab initio prediction of the three-dimensional structure of a de novo designed protein: a double-blind case study. *Proteins*.

[14] Wu S, Skolnick J, Zhang Y (2007). Ab initio modeling of small proteins by iterative TASSER simulations. *BMC Biology*.

[15] Mukherjee S, Szilagyi A, Roy A, Zhang Y (2011). Genome-wide protein structure prediction. *Biomedical and Life Sciences*.

[16] Ewing RM, Chu P, Elisma F (2007). Large-scale mapping of human protein-protein interactions by mass spectrometry. *Molecular Systems Biology*.

[17] Uetz P, Glot L, Cagney G (2000). A comprehensive analysis of protein-protein interactions in *Saccharomyces cerevisiae*. *Nature*.

[18] Ito T, Chiba T, Ozawa R, Yoshida M, Hattori M, Sakaki Y (2001). A comprehensive two-hybrid analysis to explore the yeast protein interactome. *Proceedings of the National Academy of Sciences of the United States of America*.

[19] Venkatraman V, Yang YD, Sael L, Kihara D (2009). Protein-protein docking using region-based 3D Zernike descriptors. *BMC Bioinformatics*.

[20] Mosca R, Pons C, Fernández-Recio J, Aloy P (2009). Pushing structural information into the yeast interactome by high-throughput protein docking experiments. *PLoS Computational Biology*.

[21] Pearson WR (1990). Rapid and sensitive sequence comparison with FASTP and FASTA. *Methods in Enzymology*.

[22] Bordoli L, Kiefer F, Arnold K, Benkert P, Battey J, Schwede T (2009). Protein structure homology modeling using SWISS-MODEL workspace. *Nature Protocols*.

[23] Arnold K, Bordoli L, Kopp J, Schwede T (2006). The SWISS-MODEL workspace: a web-based environment for protein structure homology modelling. *Bioinformatics*.

[24] Karplus K (2009). SAM-T08, HMM-based protein structure prediction. *Nucleic Acids Research*.

[25] Wu S, Skolnick J, Zhang Y (2007). Ab initio modeling of small proteins by iterative TASSER simulations. *BMC Biology*.

[26] Zhang Y (2008). I-TASSER server for protein 3D structure prediction. *BMC Bioinformatics*.

[27] Laskowski RA, MacArthur MW, Moss DS, Thornton JM (1993). PROCHECK: a program to check the stereochemical quality of protein structures. *Journal of Applied Crystallography*.

[28] Lovell SC, Davis IW, Arendall WB (2003). Structure validation by C*α* geometry: *φ*, *ψ* and C*β* deviation. *Proteins*.

[29] Ritchie DW, Kemp GJL (2000). Protein docking using spherical polar Fourier correlations. *Proteins*.

[30] Macioszek J, Anderson JB, Anderson LE (1990). Isolation of chloroplastic phosphoglycerate kinase: kinetics of the two-enzyme phosphoglycerate kinase/glyceraldehyde-3-phosphate dehydrogenase couple. *Plant Physiology*.

[31] Raines CA, Paul MJ (2006). Products of leaf primary carbon metabolism modulate the developmental programme determining plant morphology. *Journal of Experimental Botany*.

[32] Smith AM, Stitt M (2007). Coordination of carbon supply and plant growth. *Plant, Cell and Environment*.

[33] Steven CH, Joan LH (1992). Role of sucrose-phosphate synthase in sucrose metabolism in leaves. *Plant Physiology*.

[34] Cai Y, Xie D, Wang Z, Hong M (2002). Interaction of rice bZIP protein REB with the 5′-upstream region of both rice sbe1 gene and waxy gene. *Chinese Science Bulletin*.

[35] Raines CA (2011). Increasing photosynthetic carbon assimilation in C_3_ plants to improve crop yield: current and future strategies. *Plant Physiology*.

[36] Banks FM, Driscoll SP, Parry MAJ (1999). Decrease in phosphoribulokinase activity by antisense RNA in transgenic tobacco. Relationship between photosynthesis, growth, and allocation at different nitrogen levels. *Plant Physiology*.

[37] Raines CA (2003). The Calvin cycle revisited. *Photosynthesis Research*.

[38] Stitt M, Lunn J, Usadel B (2010). Arabidopsis and primary photosynthetic metabolism—more than the icing on the cake. *Plant Journal*.

